# Case report: a special case of cryptococcal infection-related inflammatory syndrome in a non-HIV infected and non-transplant patient

**DOI:** 10.1186/s12883-022-02773-4

**Published:** 2022-07-06

**Authors:** Xiao-Hong Su, Wei-Peng Li, Qi Lin, Xiu-Jun Zheng, Ting Fang, Ying Jiang, Fu-Hua Peng

**Affiliations:** 1 Department of Neurology, The Third Affiliated Hospital of Sun Yat-sen University, Guangzhou, China; 2 Department of Neurology, Integrated Hospital of Traditional Chinese Medicine, Southern Medical University, Guangzhou, China; 3 Department of Neurology, Shantou Central Hospital, Shantou, China

**Keywords:** Cryptococcal meningoencephalitis, Infection-related inflammatory syndrome, Post-infectious inflammatory response syndrome, Immune reconstitution inflammatory syndrome, Magnetic resonance imaging, Metagenomic next-generation sequencing

## Abstract

**Background:**

Cryptococcal meningoencephalitis (CM) is a severe infection of central nervous system with high mortality and morbidity. Infection-related inflammatory syndrome is a rare complication of CM. Herein, we report a case of CM complicated by infection-related inflammatory syndrome.

**Case presentation:**

A 42-year-old man with chronic hepatitis B presented with a 3-day history of aphasia and left hemiparesis at an outside medical facility. The brain magnetic resonance imaging (MRI) showed symmetric and confluent hyperintense signal abnormalities mainly located in the basal ganglia, internal capsule, external capsule, periventricular, corona radiata, frontal and temporal lobes. Cerebrospinal fluid (CSF) examinations revealed elevated leukocyte and protein. India ink staining was positive for *Cryptococcus.* CSF culture and metagenomic next-generation sequencing (mNGS) confirmed *Cryptococcus neoformans.* Initial response was observed with intravenous fluconazole (400 mg per day). However, 11 days later, he developed impaired consciousness and incontinence of urine and feces. A repeat brain MRI showed the lesions were progressive and enlarged. The patient was referred to our department at this point of time. Repeat CSF analysis (India ink staining, culture and mNGS) re-confirmed *Cryptococcus*. However, clinical worsening after initial improvement, laboratory examinations and brain MRI findings suggested a diagnosis of infection-related inflammatory syndrome. Therefore, a combination of corticosteroids and antifungal therapy was initiated. At follow-up, a complete neurological recovery without any relapse was documented. The repeat brain MRI showed complete resolution of the previous lesions.

**Conclusions:**

This case demonstrated that cryptococcal inflammatory syndromes must be suspected in cases of CM if an otherwise unexplained clinical deterioration is observed after initial recovery. The same can happen even before the primary infection is controlled. Thus, timely identification and prompt treatment is vital to reduce the mortality and disability of CM. The administration of corticosteroids in combination with antifungal therapy is an effective strategy in such cases.

**Graphical abstract:**

Clinical course and treatment process of the patient. Hemiparalysis and aphasia improved after the initiation of antifungal treatment. However, the patient developed impaired consciousness companied by deterioration of brain MRI findings. He was treated with adjunctive glucocorticoid taper therapy consisting of dexamethasone (20 mg/day, intravenously) for 1 week followed by oral prednisone 1 mg/kg/day, tapered based on clinical and radiological response, along with amphotericin B (0.6 mg/kg/day, intravenously), voriconazole (400 mg/day in 2 divided doses, intravenously), and 5-flucytosine (100 mg/kg/day in 4 divided doses, orally). Two weeks later, his symptoms improved significantly. After discharge, he began oral voriconazole for consolidation and maintenance therapy for 8 weeks and 9 months respectively. He recovered without any neurological sequelae at 6-month follow-up. Note: MRI = magnetic resonance imaging.
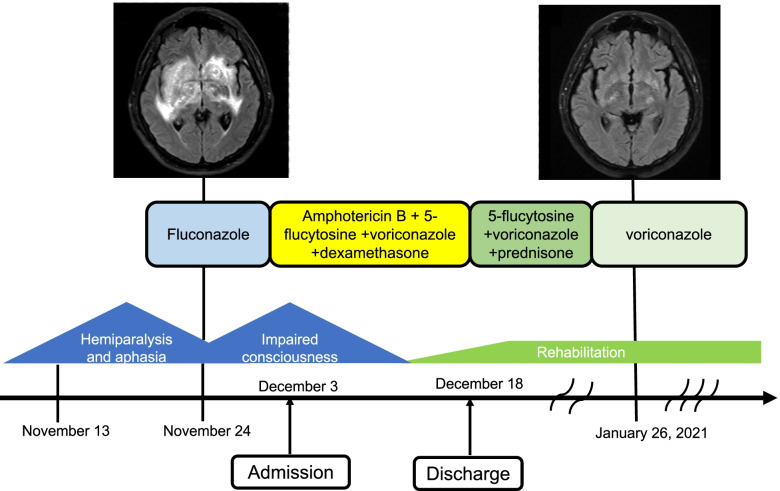

**Supplementary Information:**

The online version contains supplementary material available at 10.1186/s12883-022-02773-4.

## Background

Cryptococcal meningoencephalitis (CM) is a global invasive fungal infection with high morbidity, mortality and risk of neurological sequelae [1]. It is well described in HIV-positive population; however, it also occurs in an increasing number of HIV-negative patients [2]. A potentially serious complication of CM is a paradoxical inflammatory response in the setting of microbiologic control, known as immune reconstitution inflammatory syndrome in HIV-positive patients [3] or post-infectious inflammatory response syndrome (PIIRS) in immunocompetent patients [4]. This syndrome is characterized by refractory symptoms and/or clinical deterioration and can be clinically assessed according to Glasgow Coma Scores and brain magnetic resonance imaging (MRI) findings (e.g., diffuse leptomeningeal enhancement, multifocal supratentorial and infratentorial lesions) [5–8]. The median onset time of PIIRS was 50 days after the initiation of antifungal treatment and most of the signs were raised intracranial pressure (severe headache and nausea) and impaired nerve functions (vision and hearing impairment) in our previous report [9]. However, infection-related inflammatory syndrome, a clinical syndrome similar to PIIRS, is rarely reported. It is a vigorous inflammatory response characterized by clinical symptoms of worsening neurological function impairments and altered mental status in HIV-negative CM patients before fungal control.

Here, we report an HIV-negative CM patient with chronic hepatitis B and positive serum antinuclear antibody (ANA) presented with infection-related inflammatory syndrome.

## Case presentation

**Fig. 1:**
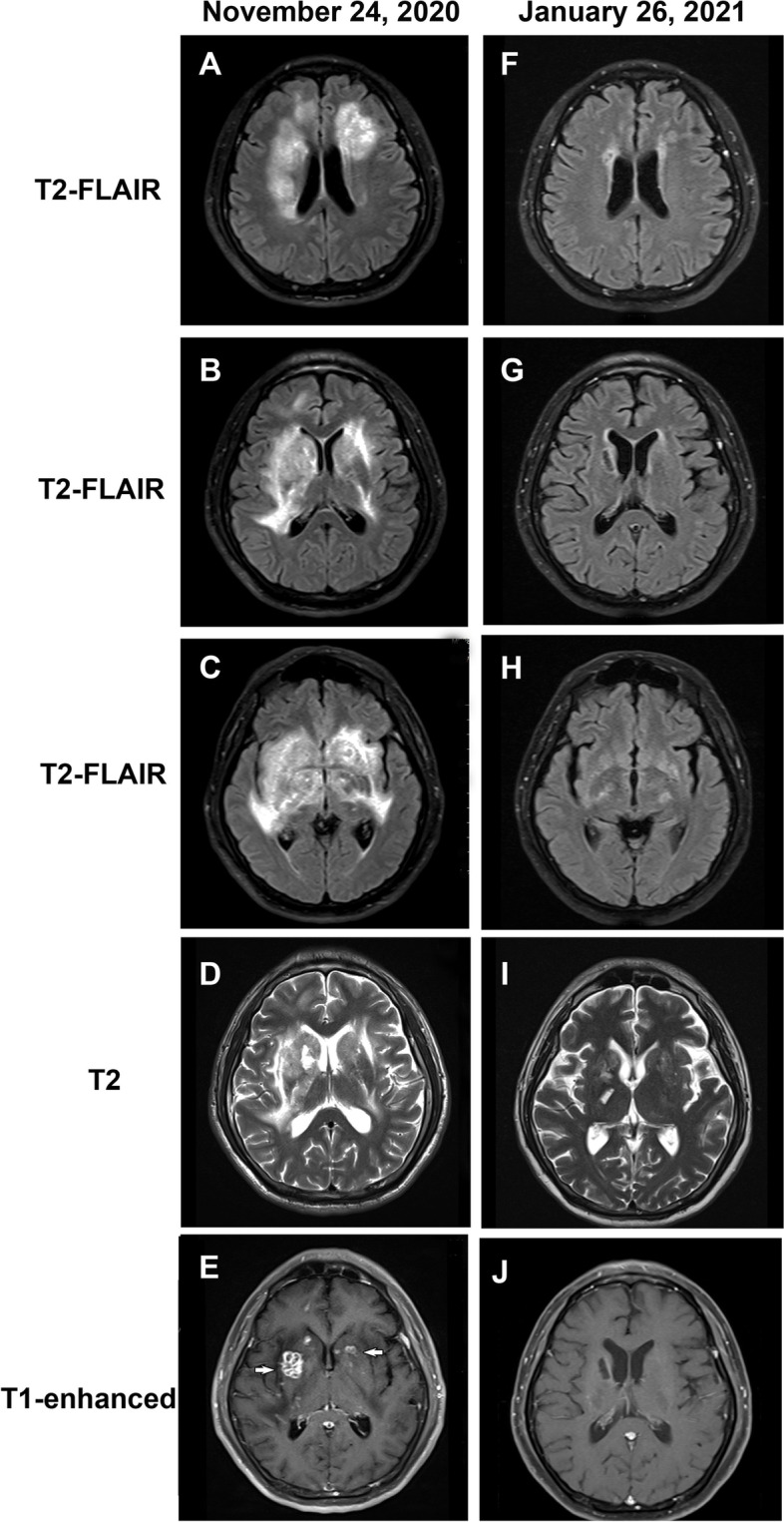
Neuroimaging features of the patient. **A**-**E** Brain MRI performed before the administration of corticosteroids. **A**-**C** T2-FLAIR images demonstrate multiple hyperintense lesions at different levels, which mainly located in the basal ganglia, internal capsule, external capsule, periventricular, corona radiata, frontal and temporal lobes. **D** Conventional T2-weighted image shows multiple lesions with poorly defined boundaries in the bilateral basal ganglia. **E** Gadolinium-enhanced T1-weighted image shows multiple ring-shaped zones of peripheral contrast enhancement in the right basal ganglia and nodular contrast enhancement in the bilateral basal ganglia (white arrows). **F**-**J** Brain MRI performed on the day 54 after treatment with corticosteroids. **F**-**H** T2-FLAIR images show almost complete disappearance of previous lesions (**I**) Conventional T2-weighted image shows that lesions in the bilateral basal ganglia were significantly reduced. **J** Gadolinium-enhanced T1-weighted image shows most of the contrast-enhancing lesions disappeared. Note: MRI = magnetic resonance imaging; FLAIR = fluid-attenuated inversion recovery

A 42-year-old Chinese Han male patient with chronic hepatitis B presented with a 3-day history of receptive aphasia, left hemiparesis and swallowing difficulties at an outside medical facility. Except for chronic hepatitis B (hepatitis virus B deoxyribonucleic acid [HBV DNA] =547 IU/mL), his medical history was normal. On the second day of admission, the brain MRI showed symmetric and confluent hyperintense signal abnormalities mainly located in the basal ganglia, internal capsule, external capsule, periventricular, corona radiata, frontal and temporal lobes (Figure S1A-E). The cerebrospinal fluid (CSF) examination revealed normal intracranial pressure (100 mmH2O), elevated leukocyte (737/μL) and protein (0.94 g/L). Routine chemistry tests were all normal except for elevated transaminase (alanine aminotransferase 289 U/L). Autoimmune serology revealed positive ANA with a titer of 1:320. Empirically, he received both anti-bacterial and antiviral treatment. However, India ink staining was positive for *Cryptococcus*. CSF culture and metagenomic next-generation sequencing (mNGS) confirmed *Cryptococcus neoformans*. The diagnosis of CM was confirmed, and his condition improved while receiving intravenous fluconazole (intravenous, 400 mg per day). Entecavir was also administered at 0.5 mg per day to prevent hepatitis B virus reactivation. However, 11 days later, he developed impaired consciousness and incontinence of urine and feces. A repeat brain MRI revealed the lesions were progressive and enlarged (Fig. 1A-E). Then the patient was transferred to our department.

On admission to our hospital, electroencephalography revealed diffuse delta waves without electrographic seizures. Repeat CSF analysis revealed intracranial pressure of 230 mmH2O, leukocyte count of 80/μL and protein concentration of 0.7 g/L. Additionally, India ink staining revealed a fungal count of 5/mL (Figure S2A), and CrAg titers in CSF and serum were 1:1 and 1:40, respectively (Figure S2B). Furthermore, *Cryptococcus neoformans* was re-identified by CSF mNGS (Figure S2C, D). Meanwhile, CSF culture was also positive for *Cryptococcus neoformans.* CSF Gram staining and acid-fast staining and serum HIV antibody were negative. And the tests for hepatitis virus A, hepatitis virus C and hepatitis virus E were negative. In addition, the serum and CSF were negative for autoimmune encephalitis-related antibodies. The flow cytometry of CSF showed normal immunophenotype and percentages of immune cells (Figure S3). Whole-body positron emission tomography (PET) scan revealed no evidence of underlying malignancy.

Based on the above MRI and CSF findings, cryptococcal infection-related inflammatory syndrome was diagnosed. Therefore, a combination of corticosteroids and antifungal therapy was initiated. The corticosteroids therapy consisted of dexamethasone (20 mg/day, intravenously) for 1 week followed by oral prednisone 1 mg/kg/day, tapered based on clinical and radiological response. The antifungal therapy consisted of amphotericin B (0.6 mg/kg/day, intravenously), voriconazole (400 mg/day in 2 divided doses, intravenously), and 5-flucytosine (100 mg/kg/day in 4 divided doses, orally). After 1 week of treatment, his condition improved and a repeat MRI showed partial improvement of the hyperintense signal lesions (Figure S1F-J). Two weeks later, his symptoms improved significantly. After discharge, he began oral voriconazole for consolidation and maintenance therapy for 8 weeks and 9 months respectively. Brain MRI was repeated on the 54 days of corticosteroids treatment (January 26, 2021), and most of the hyperintense signal lesions disappeared (Fig. 1F-J). Repeat CSF culture was negative. No neurological sequelae were observed at 6-month follow-up.

## Discussion and conclusions

CM can cause significant morbidity and mortality in HIV-negative patients, with an estimated mortality 10–30% [10–12]. Immune reconstitution inflammatory syndrome is well described in HIV-positive patients [13]. Similarly, cryptococcal PIIRS in HIV-negative CM patients is also an immune reconstitution resulting from an interaction between *Cryptococcus* and host factors. *Cryptococcus* infection had a local immune suppression effect, which may lead to a subsequent partial immune reconstitution after effective antifungal treatment [14–16].

According to our previously published paper [9], PIIRS occurred in HIV-negative CM patients with a median of 50 days after the initiation of antifungal treatment, and most of patients suffered from severe headache, nausea, vision and hearing impairment. And, all patients had negative CSF fungal cultures at PIIRS diagnosis. Another study reported that the most frequent clinical manifestations at PIIRS diagnosis were altered mental status and vision changes [17]. The diagnosis of CM was confirmed in our case, and he probably developed infection-related inflammatory syndrome 4 days after symptom onset based on the brain MRI and inflammatory CSF findings. However, the initial diagnosis and treatment of infection-related inflammatory syndrome were delayed due to his atypical signs (no headache and no raised intracranial pressure). Despite appropriate antifungal treatment, his mental status continued to decline with a related radiological deterioration on brain MRI. The monitoring of brain MRI and positive fungal culture supported our consideration of infection-related inflammatory syndrome. Subsequently, combined treatment with corticosteroids resulted in rapid clinical and radiological improvement. The positive fungal culture suggested active *Cryptococcus* infection, indicating that infection-related inflammatory syndrome occurred before microbiological control. The evolution of the disease revealed the interaction between infection and inflammation. After corticosteroids treatment, a follow-up brain MRI revealed the lesions almost completely disappeared and leptomeningeal enhancement was significantly improved.

In CM patients, it is increasingly reported that administration of corticosteroids can suppress pathological inflammatory responses, control cerebral edema, and improve clinical outcomes [14, 17–19]. Mehta et al. reported that corticosteroid salvage therapy in 13 HIV-negative patients with cryptococcal PIIRS can result in neurological improvement [18]. Anjum et al. reported that adjunctive pulse corticosteroid taper therapy could improve clinical outcomes in 15 previously healthy patients with cryptococcal PIIRS [17]. These results are consistent with our previous observations that administration of corticosteroids was associated with lower rates of fever and better modified Rankin Score scores in cryptococcal PIIRS [20]. Corticosteroids may directly affect signal transduction pathways and exert their anti-inflammatory effects by multiple mechanisms [21, 22]. Infection-related inflammatory syndrome, a clinical syndrome similar to PIIRS, also is a vigorous inflammatory response. According to the treatment response of this case, corticosteroids can exert anti-inflammatory effect similar to PIIRS. Furthermore, this case described the application of corticosteroids in cryptococcal infection-related inflammatory syndrome with chronic hepatitis B. Long-term careful monitoring of transaminases would be required because the corticosteroid treatment might enhance replication of HBV DNA, aggravate the condition, and lead to fulminant liver failure. Therefore, this case also received entecavir to suppress HBV replication. In addition, the positive ANA indicates that there may be hidden immune system abnormalities in this case, which will make him more vulnerable to *Cryptococcus* infection.

However, there are no unified guidelines on corticosteroids treatment in CM patients with infection-related inflammatory syndrome, and the treatment strategy usually depends on the disease status of each individual. In CM, microbe-mediated damage due to fungal proliferation or host-mediated damage due to excessive inflammation can lead to brain damage. The balance between host and pathogens determines the nature of the disease process. However, the treatment of cryptococcal infection-related inflammatory syndrome faces many challenges because of the need to balance immune responses and microbial control during the treatment. Clinicians do not usually know whether the preponderance of damage is due to host factors, microbial factors, or both. Without timely treatment, the inflammation can rapidly lead to clinical worsening, with mortality rates of 16–44% in HIV-negative CM patients [23]. Clinicians should be able to recognize variable clinical and neuroimaging features and choose appropriate management.

Early diagnosis and treatment are necessary because cryptococcal infection-related inflammatory syndrome can lead to severe complications and worsen the prognosis of CM patients. In this case, corticosteroids play an important role in modulating hyperinflammatory symptoms and improving clinical outcomes.

## Supplementary Information

**Additional file 1: Figure S1.** Neuroimaging features of the patient. (A-E) Brain MRI performed on Day 4 after initial symptom onset. (A-C) T2-FLAIR images demonstrate multiple hyperintense lesions at different levels, which mainly located in the basal ganglia, internal capsule, external capsule, periventricular, corona radiata, frontal and temporal lobes. (D) Conventional T2-weighted image shows multiple lesions with poorly defined boundaries in the bilateral basal ganglia. (E) T1-weighted image shows hypointense lesions with poorly defined boundaries in the bilateral basal ganglia. (F-J) Brain MRI performed after 1 week of corticosteroids treatment. (F-H) T2-FLAIR images reveal partial improvement of the hyperintense signal lesions. (I) Conventional T2-weighted image shows that lesions in the bilateral basal ganglia were slightly improved. (J) Gadolinium-enhanced T1-weighted image shows partial improvement of previous contrast-enhancing lesions. Note: MRI = magnetic resonance imaging; FLAIR = fluid-attenuated inversion recovery.

**Additional file 2: Figure S2.** (A) India ink staining of CSF shows typical round encapsulated *Cryptococcus neoformans.* (B) CrAg testing of the cerebrospinal fluid and serum reveals positive results, scale bar 50 μm. (C and D) The DNA strictly map read number for *Cryptococcus neoformans* in this patient. Note: CSF = cerebrospinal fluid; CrAg = Cryptococcal antigen; DNA = deoxyribonucleic acid.

**Additional file 3: Figure S3.** Flow cytometry of cerebrospinal fluid show normal immunophenotypes in the patient.

## Data Availability

The data sets used during the current study are available from the corresponding authors on reasonable request. Further inquiries can be directed to the corresponding author.

## Abbreviations

CM: Cryptococcal meningoencephalitis
CSF: Cerebrospinal fluid
DNA: Deoxyribonucleic acid
HIV: Human immunodeficiency virus
HBV: Hepatitis B virus
mNGS: Metagenomic next-generation sequencing
CrAg: Cryptococcal antigen
MRI: Magnetic resonance imaging
PIIRS: Post-infectious inflammatory response syndrome
