# Supplementary figures and images for: Case report: a special case of cryptococcal infection-related inflammatory syndrome in a non-HIV infected and non-transplant patient
Source: BMC Neurol. 2022 Jul 6;22:247. doi: 10.1186/s12883-022-02773-4 (PMC9258111; doi:10.1186/s12883-022-02773-4)

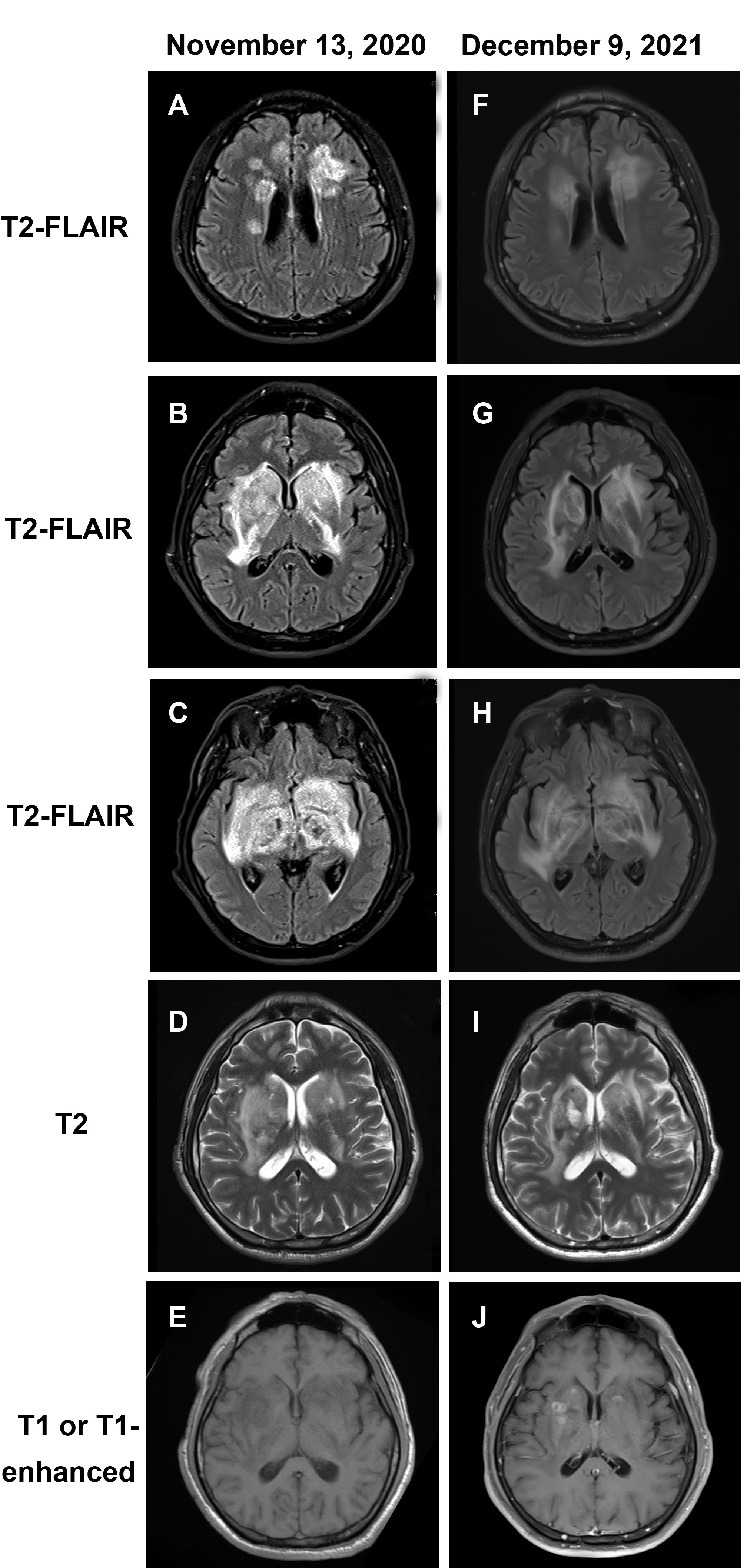

Supplement: Supplementary file 1 — Additional file 1: Figure S1. Neuroimaging features of the patient. (A-E) Brain MRI performed on Day 4 after initial symptom onset. (A-C) T2-FLAIR images demonstrate multiple hyperintense lesions at different levels, which mainly located in the basal ganglia, internal capsule, external capsule, periventricular, corona radiata, frontal and temporal lobes. (D) Conventional T2-weighted image shows multiple lesions with poorly defined boundaries in the bilateral basal ganglia. (E) T1-weighted image shows hypointense lesions with poorly defined boundaries in the bilateral basal ganglia. (F-J) Brain MRI performed after 1 week of corticosteroids treatment. (F-H) T2-FLAIR images reveal partial improvement of the hyperintense signal lesions. (I) Conventional T2-weighted image shows that lesions in the bilateral basal ganglia were slightly improved. (J) Gadolinium-enhanced T1-weighted image shows partial improvement of previous contrast-enhancing lesions. Note: MRI = magnetic resonance imaging; FLAIR = fluid-attenuated inversion recovery. [file 12883_2022_2773_MOESM1_ESM.jpg]

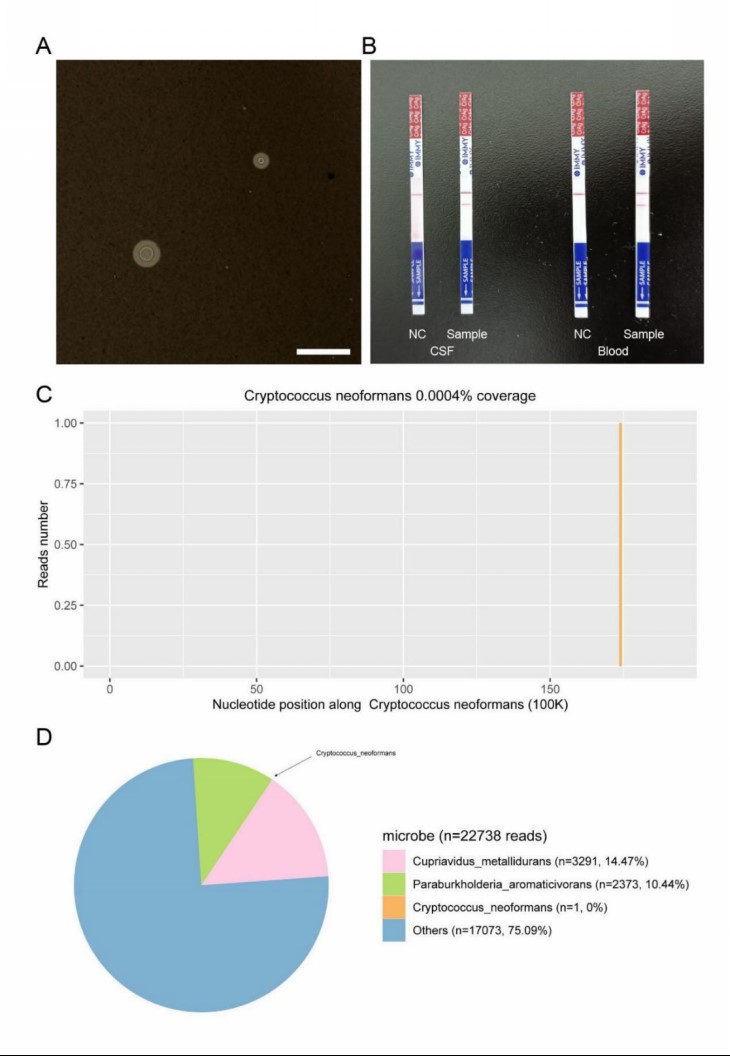

Supplement: Supplementary file 2 — Additional file 2: Figure S2. (A) India ink staining of CSF shows typical round encapsulated Cryptococcus neoformans. (B) CrAg testing of the cerebrospinal fluid and serum reveals positive results, scale bar 50 μm. (C and D) The DNA strictly map read number for Cryptococcus neoformans in this patient. Note: CSF = cerebrospinal fluid; CrAg = Cryptococcal antigen; DNA = deoxyribonucleic acid. [file 12883_2022_2773_MOESM2_ESM.jpg]

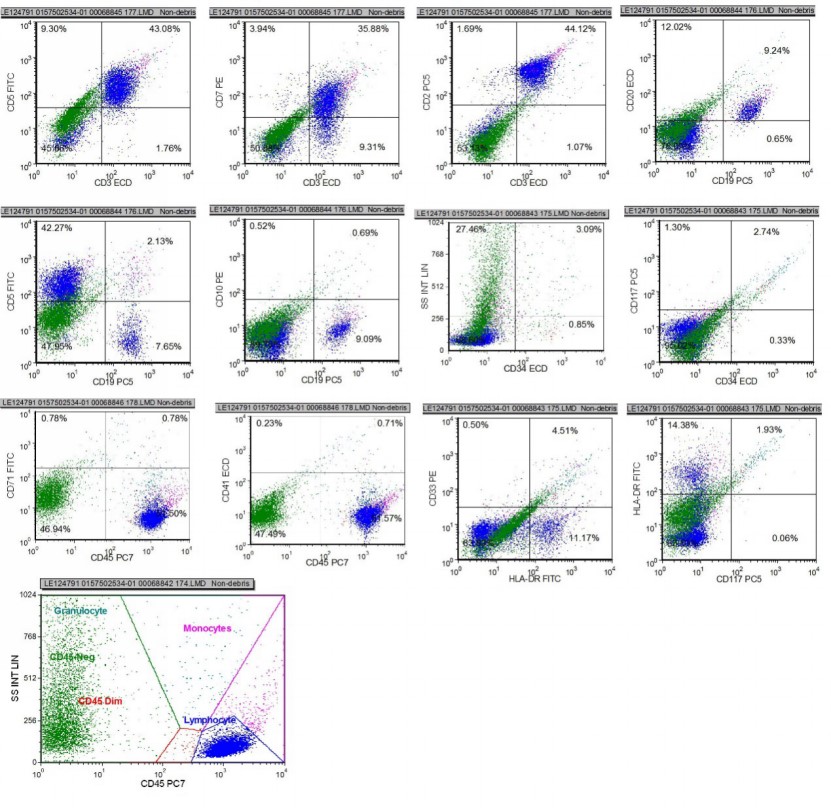

Supplement: Supplementary file 3 — Additional file 3: Figure S3. Flow cytometry of cerebrospinal fluid show normal immunophenotypes in the patient. [file 12883_2022_2773_MOESM3_ESM.jpg]
